# Onchocerciasis control in Ghana (1974–2016)

**DOI:** 10.1186/s13071-020-04507-2

**Published:** 2021-01-02

**Authors:** Nana-Kwadwo Biritwum, Dziedzom K. de Souza, Odame Asiedu, Benjamin Marfo, Uche Veronica Amazigo, John Owusu Gyapong

**Affiliations:** 1 Neglected Tropical Diseases Programme, Ghana Health Service, Accra, Ghana; 2 Bill & Melinda Gates Foundation, Seattle, USA; 3 Department of Parasitology, Noguchi Memorial Institute for Medical Research, University of Ghana, Legon-Accra, Ghana; 4 Pan-African Community Initiative on Education and Health (PACIEH), Enugu, Nigeria; 5 University of Nigeria, Nsukka, Enugu Nigeria; 6 University of Health and Allied Sciences, Ho, Ghana

**Keywords:** Onchocerciasis control, Ghana, Ivermectin, Community-directed ivermectin treatment

## Abstract

**Background:**

The control of onchocerciasis in Ghana started in 1974 under the auspices of the Onchocerciasis Control Programme (OCP). Between 1974 and 2002, a combination of approaches including vector control, mobile community ivermectin treatment, and community-directed treatment with ivermectin (CDTI) were employed. From 1997, CDTI became the main control strategy employed by the Ghana OCP (GOCP). This review was undertaken to assess the impact of the control interventions on onchocerciasis in Ghana between 1974 and 2016, since which time the focus has changed from control to elimination.

**Methods:**

In this paper, we review programme data from 1974 to 2016 to assess the impact of control activities on prevalence indicators of onchocerciasis. This review includes an evaluation of CDTI implementation, microfilaria (Mf) prevalence assessments and rapid epidemiological mapping of onchocerciasis results.

**Results:**

This review indicates that the control of onchocerciasis in Ghana has been very successful, with a significant decrease in the prevalence of infection from 69.13% [95% confidence interval) CI 60.24–78.01] in 1975 to 0.72% (95% CI 0.19–1.26) in 2015. Similarly, the mean community Mf load decreased from 14.48 MF/skin snip in 1975 to 0.07 MF/skin snip (95% CI 0.00–0.19) in 2015. Between 1997 and 2016, the therapeutic coverage increased from 58.50 to 83.80%, with nearly 100 million ivermectin tablets distributed.

**Conclusions:**

Despite the significant reduction in the prevalence of onchocerciasis in Ghana, there are still communities with MF prevalence above 1%. As the focus of the GOCP has changed from the control of onchocerciasis to its elimination, both guidance and financial support are required to ensure that the latter goal is met.
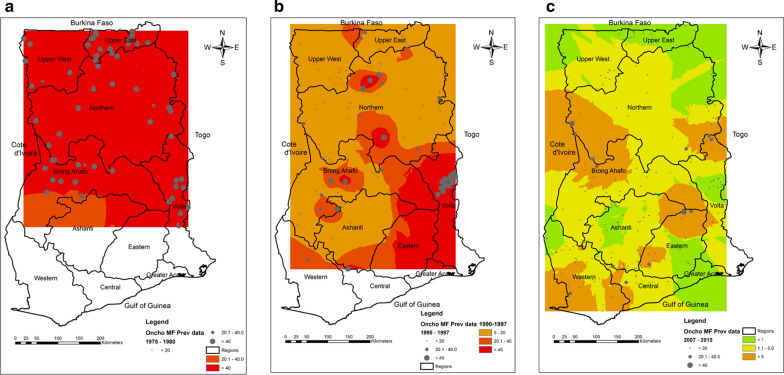

## Background

Onchocerciasis is a neglected tropical disease (NTD) caused by the nematode *Onchocerca volvulus*, which is transmitted by the black fly [1]. The disease is endemic in 28 sub-Saharan countries in Africa including Ghana, in Yemen, and in small foci in six Latin American countries [2]. Onchocerciasis is highly debilitating, giving rise to severe visual loss and blindness and severe itching with dermal changes [3]. The disease is often referred to as ‘river blindness’ because the populations that are worst affected live close to fast-flowing rivers where the vectors breed. In these highly endemic communities, the disease has a serious socio-economic impact [4]. The WHO’s implementation of the Onchocerciasis Control Programme (OCP) in West Africa from 1974 to 2002, followed by the African Programme for Onchocerciasis Control (APOC) from 1995 to 2015, has led to a substantial reduction of the disease burden [5].

Ghana is one of the West African countries endemic for onchocerciasis, and has been involved in the control of onchocerciasis since the inception of the OCP. During the OCP (1974–2002), vector control, through aerial larviciding, was implemented in the northern and central parts of the country [6]. Following the licensing of ivermectin for human use [7, 8], Ghana became one of the first countries to implement mass drug administration, with trials undertaken in some highly endemic foci [9]. From 1992 to 1997, ivermectin was distributed in Ghana by mobile teams. The goal of the Ghana OCP (GOCP) then was to reduce the prevalence of onchocerciasis to such a low level that it would cease to be of concern for public health. Thus, in 1997, Ghana piloted community-directed treatment with ivermectin (CDTI) as its main strategy for the control of onchocerciasis, which was then upscaled in 1998 [10]. However, the recent change in the global strategy for onchocerciasis from control to elimination [11] means that the operations of the Ghana programme need to be adapted to meet this aim. This review was undertaken to assess the progress made in Ghana during the control phase and to identify the challenges and requirements for future activities of the GOCP.

## Methods

Onchocerciasis data were obtained from the Ghana NTD programme, which is responsible for all the activities in relation to onchocerciasis control in Ghana. The data were compiled for various activities from the inception of the OCP in 1974 until 2016. This review includes an evaluation of CDTI implementation, microfilaria (Mf) prevalence assessments, rapid epidemiological mapping of onchocerciasis (REMO) results, and entomological assessments, where available. Activities from 2017 to date, which include a major impact assessment, are considered in a different publication since these form part of the elimination activities of the programme.

### Data analysis

The programme data were compiled in Microsoft Excel and the graphs generated in GraphPad Prism (version 6.05). Other statistical analyses were undertaken using MedCalc Software (version 18.6). Statistical significance was determined at* p*-values < 0.05. The prevalence of infection is presented as the percentage of surveyed participants with nodules or Mf in skin snips, together with the 95% confidence intervals (CIs). Due to the fact that the Mf prevalence data were not systematically collected in all endemic communities and in the same years, community- and district-level analyses were not always possible. The mean intensity of infection or community Mf load (CMFL) was estimated where possible. Entomological surveillance data are only presented for those sites for which there were complete datasets.

During the programme, the GPS coordinates of surveyed communities were recorded for each village using a hand-held Garmin Global Positioning System (GPS) unit. Where the coordinates were not available, the locations of the communities were geo-referenced using the latitude and longitude coordinates obtained by cross-checking their names with data from the Directory of Cities and Towns in the World [12] database. The data were imported into the geographical information system (GIS) software ArcGIS 10.2 (ESRI, Redlands, CA) for mapping. Due to the small number of communities assessed in some years, the data were grouped into year periods. For each of these periods, the mean prevalence was estimated. Based on the data points, the distribution of the disease in Ghana was modelled through kriging analysis in ArcGIS.

## Results

### Vector control and surveillance

Onchocerciasis vector control in Ghana was undertaken as part of the OCP between 1974 and 2002. Vector control activities were mainly focused on the northern and central savannah areas of the country [6]. Thus, the southern forest foci were not included in the OCP activities. In these areas, several entomological investigations were conducted between 2009 and 2011 [13–15]. Despite repeated ivermectin treatment, transmission was detected in all the communities assessed, and in two of these communities the thresholds were above the WHO recommendations of one infective stage-three (L3) larva per 1000 parous flies [14, 16]. The GOCP also conducted entomological surveillance in different years of the programme stages, albeit with incomplete data since most of the analyses were done outside the country. Available and complete data for 2009 and 2010 are presented. Through the support of the APOC, fly collection was undertaken in: (i) the initial OCP area where control activity was exclusively based on vector control; (ii) in the OCP southern extension (central savannah) area where both vector control and ivermectin treatment were used; and (iii) in the forest areas where control activities were based exclusively on ivermectin treatment. Collected flies were processed using the pool-screening *O. volvulus*-specific O-150 polymerase chain reaction method. The outcomes of these evaluations are presented in Table 1, and reveal that, in some sites where the minimum 6000 flies were tested, the infectivity rate was above the 1/2000 flies threshold level, which is used as an indicator for the interruption of disease transmission [16].

**Table 1:** Infectivity rates of vectors collected in Ghana (2009, 2010)

Location	Year	No. expected	No. tested	Infectivity rate (10^−3^)	Interval (10^−3^)	
Ahamasu	2009	6000	8401	0.24	0.02	0.86
Tintinso	2009	6000	4203	2.53	1	5.23
Pibila	2009	6000	3016	6.9	2.32	18.15
Bielikpong	2009	6000	11098	0.28	0.05	0.84
Nsawora	2009	6000	4141	0.24	0.007	1.3
Kamba	2009	6000	5741	0	0	0.33
Kumdi	2009	6000	4816	0	0	0.39
Chache	2009	6000	1082	0	0	1.59
Nakong	2009	6000	2409	0	0	0.79
Abua	2009	6000	4198	0	0	0.45
Ekundipe	2010	6000	3137	1.7	0.427	4.446
Ahamasu	2010	6000	10582	0.53	0.159	1.25
Kamba	2010	6000	2294	0	0	0.79
Tainso	2010	6000	6850	0.357	0.0357	1.06
Nsawora	2010	6000	2602	0.445	0.013	2.29
Bielikpong	2010	6000	3263	0.317	0.009	1.36
Pibila	2010	6000	3196	0.35	0.01	1.81
Abua	2010	6000	4981	0.45	0.0524	1.56
Nakong	2010	6000	2575	3.655	0.892	10.17
Chache	2010	6000	778	0	0	2.131

### CDTI implementation in Ghana

**Fig. 1:**
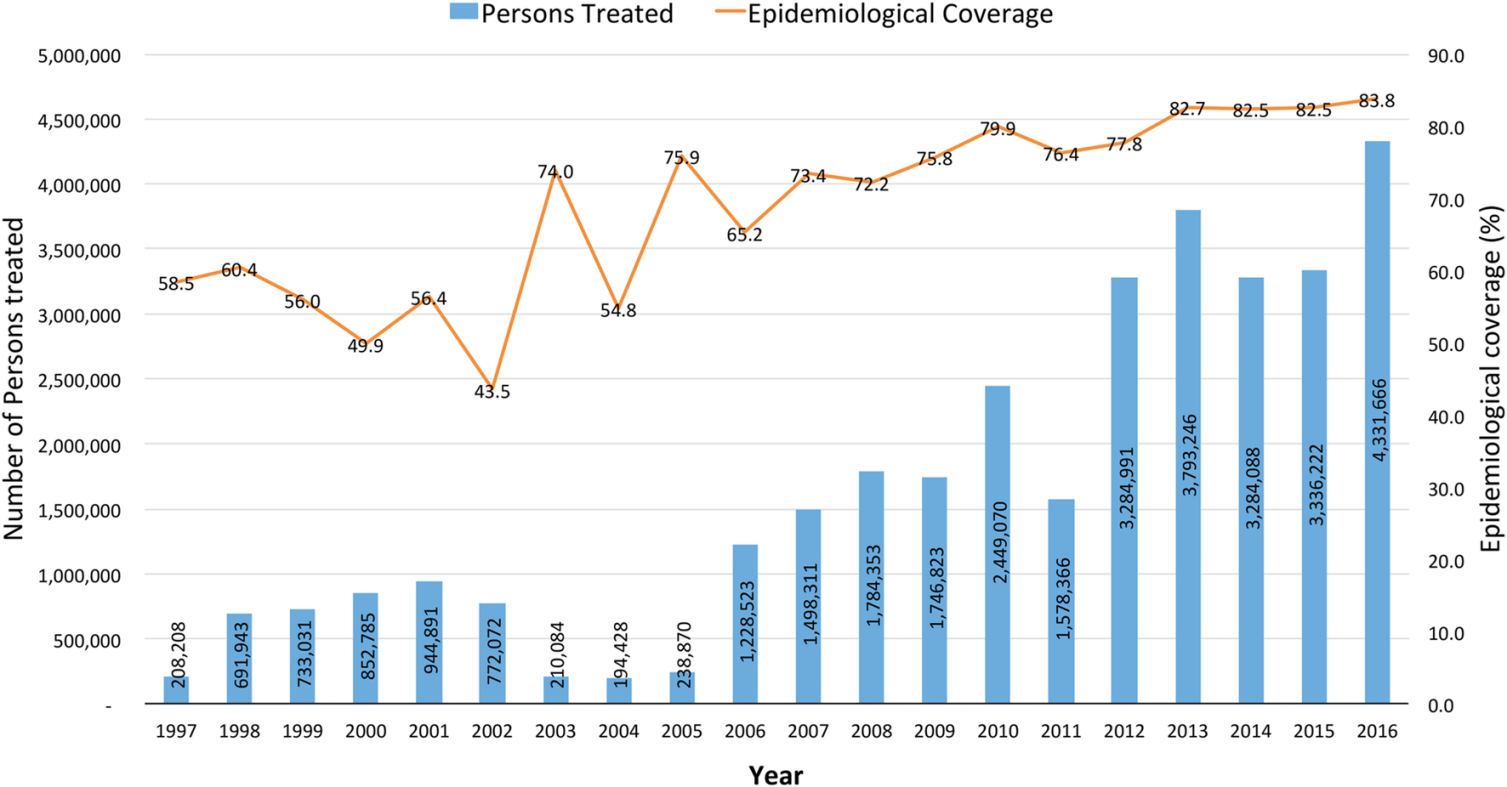
Therapeutic coverage of community-directed treatment with ivermectin in Ghana (1997–2016)

Nine out of ten regions in Ghana are onchocerciasis endemic; the CDTI strategy was implemented in all of them [10], with all endemic communities receiving treatment annually after 1998. Following the cessation of OCP operations in 2002, several communities were classified as Special Intervention Zones (SIZ; areas of hyperendemicity) between 2002 and 2007 [17], in which partial CDTI activities were undertaken. Ivermectin treatment for the elimination of lymphatic filariasis was started in 2001 and was implemented in districts co-endemic with onchocerciasis, and gradually scaled up to cover all endemic districts by 2006 [18]. The programme saw a gradual increase in the number of people treated and epidemiological coverage. In 2016, over 4 million individuals in endemic communities were treated. The therapeutic coverage increased from 58.50% in 1997 to 83.8% in 2016 (Fig 1). The coverage was consistently above 65% after 2006. Nearly 100 million ivermectin tablets were distributed within this period (Additional file 1: Fig. S1) through the onchocerciasis and lymphatic filariasis programmes.

### Remapping of onchocerciasis in Ghana

Despite the implementation of CDTI from the late 1990s, inadequate financial support coupled with management challenges led to the erratic distribution of ivermectin with poor therapeutic and geographic coverage for most of the treatment areas. The inception of the lymphatic filariasis elimination programme in 2000 led to the distribution of ivermectin and albendazole to many communities in Ghana [19]. From 2004, drug distribution as part of the lymphatic filariasis elimination programme was combined with the onchocerciasis programme, resulting in significant improvements in geographic and therapeutic coverage. However, up-to-date mapping data were not available for the country, so the onchocerciasis programme relied on historical data compiled from regional and districts health teams, as well as community surveys, to guide the treatment for onchocerciasis.

In 2008, REMO [20] was undertaken in Ghana, through the support of APOC, to accurately and rapidly re-map and identify communities suffering from onchocerciasis. Endemicity maps were developed for the preparation of a national plan for onchocerciasis control, and for a re-launch of the mass treatment of identified onchocerciasis communities with ivermectin. The selection of communities was optimally biased towards high-risk ones (those most likely to have the worst disease profile, and communities in the immediate vicinity of major potential vector breeding sites) in each endemic zone [10]. At least one high-risk community was selected in each district per stretch of the river, and along every 30- to 50-km stretch along the main river, and in the valley of each major tributary. For each of the high-risk villages, a secondary village, which was located at least 10 km further away from the breeding sites of the vectors, was chosen to help obtain some indication of the distribution and overall severity of the disease over a wider geographical area. A survey of a secondary village was only undertaken if at least some of the high-risk communities were proven to be meso- or hyperendemic for onchocerciasis. A measure of the level of endemicity of onchocerciasis in the selected communities was undertaken using the rapid epidemiological assessment (REA) method based on nodule prevalence after nodule search in a sample of adults [20].

In each community, a random sample of 50 adult males and females aged more than 20 years, and resident in the community for at least 10 years, was taken. These individuals were examined for the presence of onchocercal nodules. Two skin snip biopsies were taken from the iliac crest of each participant, and after a drop of normal saline had been added the strips were examined under a microscope after 30 min of incubation [21]. Validation of the results was undertaken within 2 months of the completion of the surveys by a team which included a group of independent experts, which surveyed a sample of villages where the original rapid assessments had been conducted. The validation of the results of these villages involved the selection of a stratified random sample that took into account the different ecological zones and levels of reported onchocercal endemicity. The demographic characteristics of the villages and the contact details of village heads were also recorded.

Out of 753 villages selected, only 705 were examined using REA (Table 2) due to accessibility. The survey also combined nodule examination with skin snip assessments in 85 villages. A total of 27,635 people were surveyed by REMO, of whom 1091 (4.0%) were positive for nodules. This proportion varied from 0.3% in the Greater Accra Region to 8.0% in the Central Region. Of the 3966 inhabitants of the 85 villages for whom skin snips were examined, 365 (9.2%) were found to be positive for Mf, with prevalence varying from 0.0% in the Greater Accra Region to 16.3% in the Ashanti Region. A case search for blindness revealed a total of 1,229 blind individuals. The Northern and Upper East Regions had the highest number of cases of blindness, 356 and 364 respectively, which comprised 58.2% of all the cases. Greater Accra had the fewest, with three cases of blindness. Community nodule prevalence rates varied from 0.0% in many regions to 59.4% in one community in the Ashanti Region, while community Mf prevalence also varied from 0.0% in many communities across the country to 53.1%, also in one community in the Ashanti Region.

**Table 2:** Summary of onchocerciasis mapping survey results by region (2008)

Region	Nodule examination				Skin snips				Blindness cases
	REMO villages	No. examined	No. positive	Nodule prevalence (%)	REMO villages (%)	No. examined	No. Mf positive	Mf prevalence	Number
Ashanti	83	3005	140	4.66 (3.93–5.47%)	11 (13)	679	111	16.35 (13.64–19.35%)	55
Brong Ahafo	86	3268	115	3.52 (2.91–4.21%)	6 (7)	244	2	0.82 (0.10–2.93%)	87
Central	26	1020	82	8.04 (6.44–9.88%)	14 (54)	501	95	18.96 (15.62–22.67%)	47
Eastern	118	4772	163	3.42 (2.92–3.97%)	6 (5)	464	51	10.99 (8.29–14.20%)	89
Greater Accra	12	341	1	0.29 (0.01–1.62%)	2 (17)	106	0	0.00 (0.00–3.42%	3
Northern	120	4741	249	5.25 (4.63–5.93%)	10 (8)	394	53	13.45 (10.24–17.22%)	356
Upper East	22	718	16	2.23 (1.28–3.59%)	6 (27)	233	0	0.00 (0.00–1.57%)	364
Upper West	46	1869	47	2.51 (1.85–3.33%)	7 (16)	307	14	4.46 (2.52–7.53%)	64
Volta	68	2882	134	4.65 (3.91–5.48%)	9 (13)	450	18	4.00 (2.39–6.25%)	106
Western	124	4987	144	2.89 (2.44–3.39%)	14 (11)	588	21	3.57 (2.22–5.41%)	58
Total	705	27603	1091	3.95 (3.73–4.19%)	85 (12)	3966	365	9.20 (8.32–10.15%)	1229

*REMO* Rapid epidemiological mapping of onchocerciasis,* Mf* microfilaria

The total number of cases of blindness identified in the Northern and Upper East regions (58.6%) gives a possible indication of the severity of onchocerciasis and other blinding diseases in the northern parts of the country. These data support earlier observations which led the former OCP to focus on onchocerciasis in the northern parts of the country, which were well known for cases of blinding onchocerciasis [6]. While onchocerciasis could have been a contributory factor to old cases of blindness, trachoma might have been the main contributory factor to new cases of preventable blindness in the northern parts of Ghana [22, 23].

### Reorganization of onchocerciasis treatment areas

The results of the REMO survey, together with those of previous epidemiological and entomological surveys, were used to help delineate areas of onchocerciasis endemicity in Ghana (Additional file 2: Fig. S2). The main infected river basins were found to be the Pra, Pru, Offin, Afram, Bia, Bui-Black Volta, Daka and Majimaji. Black flies are known to be have a normal flight range of 20 km within their breeding sites, though with the aid of certain climatic and physical conditions they are able to fly a distance of 400 km or more [24, 25]. Communities were therefore selected for treatment along the infected rivers but within the normal flight range (20 km) of the black fly. Within these identified infected river basins, all the communities within a 20-km radius were then identified by the programme and listed as communities endemic for onchocerciasis, for which biannual treatment with ivermectin was instituted in 2009. The number of communities treated was gradually scaled up from 2009 (Additional file 3: Fig. S3).

This re-mapping of onchocerciasis also resulted in the discovery of ivermectin-naive treatment areas, which had not been previously identified as onchocerciasis endemic in Ghana. These areas were the Afram Plains District of the Eastern region, the Ho Municipal District in the Volta Region and the Amansie Central District in the Ashanti Region.

### Introduction of twice-yearly treatment

Following the REMO survey in 2008, twice-a-year treatment with ivermectin was implemented from 2009 in all the identified hyperendemic (prevalence > 40%) and mesoendemic (prevalence between 20 and 39%) communities. Various categories of personnel at the community level, as well as health workers, were trained for drug distribution. Additional file 4: Fig. S4 shows the number of people at the community level that were trained annually for health intervention from 2009. Health workers were also trained every year (Additional file 5: Fig S5). Implementation units were re-demarcated from villages to entire sub-districts, as long as a village in that sub-district was endemic for onchocerciasis. Thus, there was an increase in the number of communities treated, from 1509 in 1998 to 7043 in 2016 (Additional file 3: Fig S3), which reflects the commitment of the programme to reach all endemic communities in the country.

### Impact of interventions on Mf prevalence

**Fig. 2:**
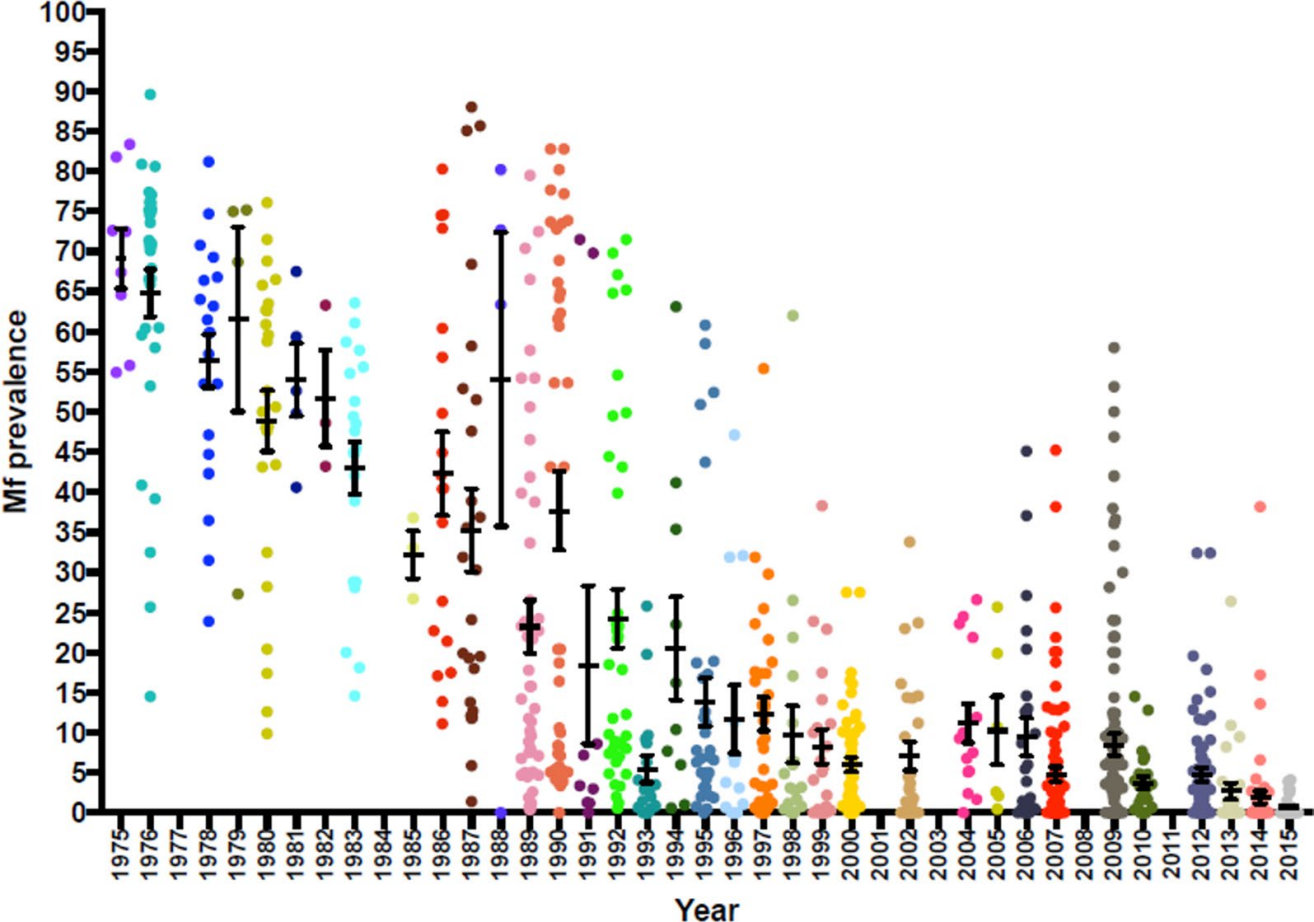
Yearly microfilaria (*Mf*) prevalence estimates. *Dots* indicate the prevalence for each community assessed.* Error bars* indicate SE,* horizontal lines* represent mean Mf prevalence

**Fig. 3:**
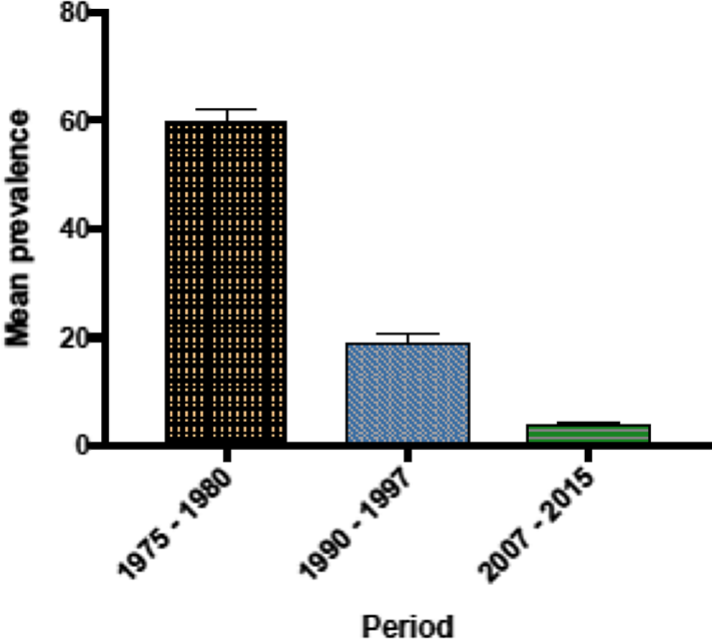
Mf prevalence estimates for the periods 1975–1980, 1990–1997 and 2007–2015.* Error bars* represent SEM

**Fig. 4:**
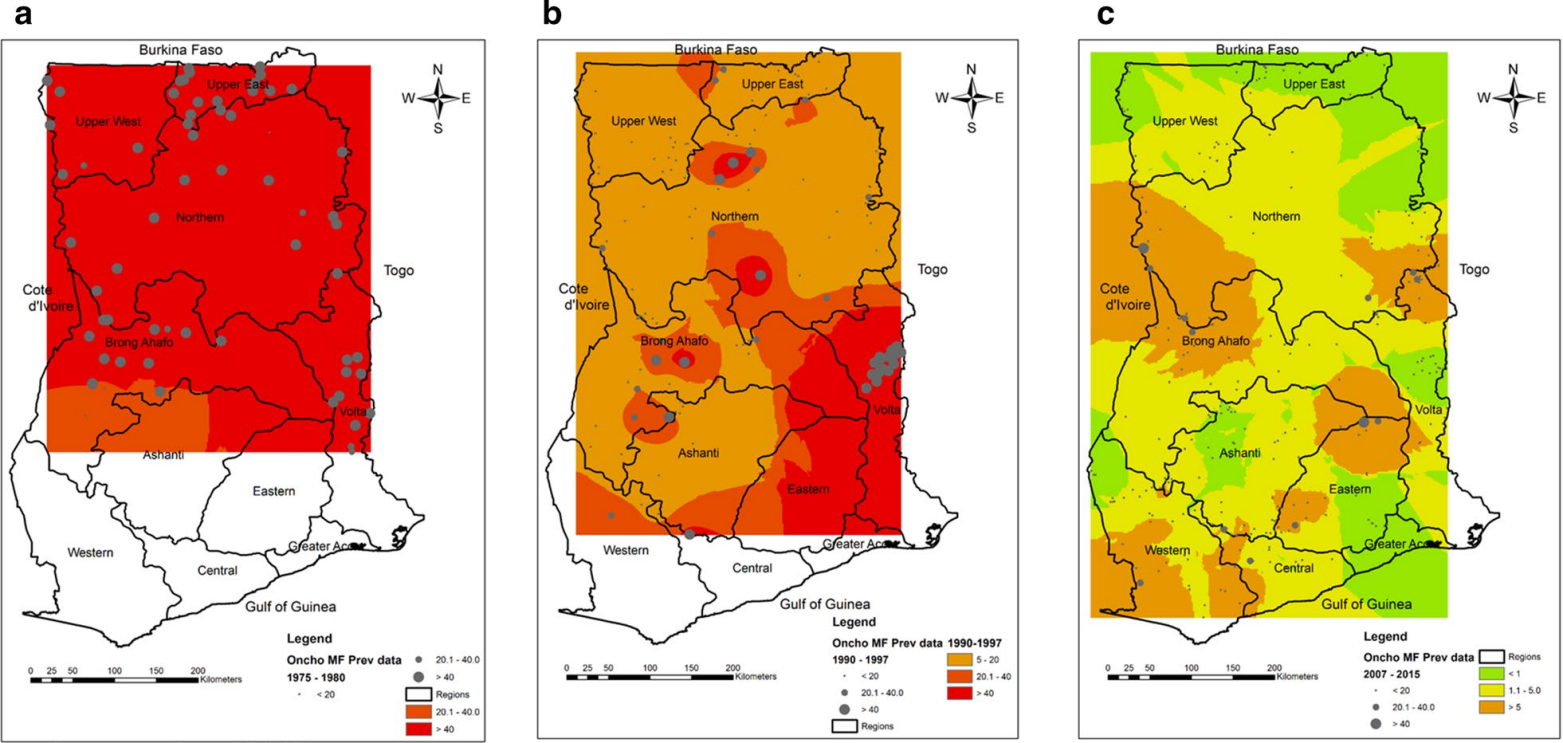
**a**–**c** Change in Mf prevalence in Ghana. **a** Hyper and mesoendemic areas, 1975–1980. **b** Hyper-, meso- and hypoendemic areas, 1990–1997; **c** classification based on elimination thresholds, 2007–2015

The prevalence data revealed a downward and significant reduction in the mean Mf prevalence from 1975 to 2015 (Figs. 2, 3). In 1975, the mean Mf prevalence was 69.13% (95% CI 60.24–78.01), with a range of 54.90–83.40% in eight surveyed communities. By 1998, when CDTI became the main control strategy, the Mf prevalence was 9.76% (95% CI 2.22–17.30), with a range of 0.00–62%. By 2015, the mean Mf prevalence decreased to 0.72% (95% CI 0.19–1.26), with a range of 0.00–4.10%, in 26 surveyed communities. The mean CMFL decreased from 14.48 MF/snip in one community in 1975 to 1.60 MF/snip (95% CI 0.00–3.93), with a range of 0.0–21.9 MF/snip in 20 communities by 1998. By 2015, the mean CMFL fell to 0.07 MF/snip (95% CI 0.00–0.19), with a range of 0.0–0.15 MF/snip in 26 surveyed communities. As a result of the CDTI activities of the programme, the status of many endemic areas changed from being hyperendemic to hypoendemic. Figure 4 shows the change in endemicity from the pre-control intervention in 1975 up until 2015. Based on the Mf results, seven transmission zones were identified. These included the Black Volta, Dayi-Asukwakwa, Oti-Daka, Pra-Offin, Pru-Afram, Tano-Ankobra and the White Volta-Kulpawn transmission zones. These transmission zones were further demarcated to identify districts that require one or two rounds of treatment per year.

## Discussion

Since its inception in 1974, the GOCP has undergone several phases of implementation with the technical and financial support of the former OCP, SIZ, APOC of the WHO, and now the WHO’s Elimination of Neglected Tropical Diseases, with strategies including vector control through aerial larviciding, mobile team distribution of ivermectin, and CDTI, together with the campaign strategy of the lymphatic filariasis programme, mainly undertaken in onchocerciasis and lymphatic filariasis co-endemic areas in Ghana. These strategies have been implemented with the technical and financial support of the United States Agency for International Development, various non-governmental development cooperation organisations and Sightsavers. This review reveals significant reductions in the prevalence and distribution of onchocerciasis in Ghana following the interventions implemented within the review period. Many areas previously hyperendemic are now considered to be hypoendemic. However, given the change of the GOCP’s focus from control to elimination, as these areas are still endemic, they will need to be dealt with accordingly in the future.

Despite the successes of the GOCP, there are still many inherent challenges associated with onchocerciasis control in Ghana. Principally, challenges in funding, technical guidance, human resources, tools and inadequate guidelines have hampered the implementation of the GOCP. The programme has also faced competition for resources from other health programmes, and the buy-in of the government into onchocerciasis control activities has been limited. Though xenomonitoring is recommended to determine when to stop mass drug administration for onchocerciasis, there is limited technical and financial support for this. The government laboratory and research institutions do not have the required capacity for this, or they are too expensive, which means that these activities are not operationally feasible. Furthermore, many of the laboratory technicians with the necessary expertise for onchocerciasis surveys are at retirement age, and younger laboratory technicians show little or no interest in onchocerciasis field surveys. A recrudescence of onchocerciasis in certain endemic areas as a result of sub-optimal responses to ivermectin treatment [26, 27] has also been observed. In these areas, the strategy of twice-yearly treatment has reduced the Mf intensity and prevalence [28]. However, Mf still remains a challenge in a number of communities.

Despite a significant reduction in the prevalence of Mf, in many communities it is still above 1%. In communities where Mf prevalence is < 1%, entomological evaluations will need to be conducted to ensure that the parasite levels within the vector remain below the threshold of one L3 larva per 1,000 parous flies [29, 30]. Furthermore, the change in strategy since 2017 from control to elimination requires re-definement of the treatment areas, determination of the impact of previous treatment, the introduction of new tools for the assessment of transmission and the elucidation of transmission zones guided by an onchocerciasis expert committee on when to stop mass drug administration. As part of this change in approach, there is a need for longitudinal onchocerciasis surveillance data, confirmed by cross-sectional survey data to elucidate the impact of past programmes and to provide further direction for programmes with the aim of elimination. The results of an onchocerciasis impact assessment survey undertaken in 2017 will be published elsewhere. However, the successful elimination of onchocerciasis requires expert technical advice, buy-in and significant funding from programme partners, a strong technical team to undertake the field surveys and an monitoring and evaluation team to manage the data and analyse and report the results to an expert committee to determine future directions towards the elimination of onchocerciasis. It is anticipated that the guidelines of the WHO Onchocerciasis Technical Advisory Subgroup [31] will help workers address some of these challenges.

## Conclusion

The significant reduction in the prevalence of onchocerciasis in many communities indicates that the control of this disease has been a success in Ghana. The success of the re-mapping of onchocerciasis to develop endemicity maps to guide the treatment of endemic communities, and the relaunched control programmes, was confirmed by a reduction in onchocerciasis endemicity. As the country moves towards the elimination of onchocerciasis, there is a need to address the following challenges: funding; human resources and technical capacity; government buy-in; possible ivermectin non-response, which has been reported in certain parts of the country [26, 32]; the financial sustainability of programme activities to achieve elimination targets; and the technical guidance of the WHO and its partners.

## Supplementary information

**Additional file 1: Fig. S1.** Cumulative number of ivermectin tablets distributed (1997–2016).

**Additional file 2: Fig. S2.** Workforce at the community level for health intervention (2009–2016).

**Additional file 3: Fig. S3.** Training of health workers (2009–2016).

**Additional file 4: Fig. S4.** Number of communities treated (1998–2016).

**Additional file 5: Fig. S5.** REMO (2008) map identifying endemic areas for treatment.

## Data Availability

The datasets used and/or analysed during the present study are available from the corresponding author on reasonable request.

## References

[1] World Health Organization. Progress report on the elimination of human onchocerciasis, 2017–2018. Rapport de situation sur l’élimination de l’onchocercose humaine, 2017–2018. Wkly Epidemiol Rec. 2018;633–43.

[2] Kim YE, Remme JHF, Steinmann P, Stolk WA, Roungou JB, Tediosi F (2015). Control, elimination, and eradication of river blindness: scenarios, timelines, and ivermectin treatment needs in Africa. PLoS Negl Trop Dis..

[3] Ahmad K (2000). WHO programme gives hope to blind and partially sighted people in Africa. Lancet..

[4] Crump A, Morel CM, Omura S (2012). The onchocerciasis chronicle: from the beginning to the end?. Trends Parasitol..

[5] WHO/APOC. Report of the external mid-term evaluation of the African programme for onchocerciasis control. JAF16.8. 2010. http://www.who.int/apoc/MidtermEvaluation_29Oct2010_final_printed.pdf. Accessed 28 Oct 2020.

[6] World Health Organization. Expert Committee on Onchocerciasis. Third report. Tech Rep Ser 752. Geneva, Switzerland; 1987. 3120423

[7] Meredith SEO, Dull HB (1998). Onchocerciasis: the first decade of Mectizan treatment. Parasitol Today..

[8] Dull HB, Meredith SEO (1998). The Mectizan donation programme—a 10-year report. Ann Trop Med Parasitol..

[9] Alley ES, Plaisier AP, Boatin BA, Dadzie KY, Remme J, Zerbo G (1994). The impact of five years of annual ivermectin treatment on skin microfilarial loads in the onchocerciasis focus of Asubende, Ghana. Trans R Soc Trop Med Hyg..

[10] Taylor MJ, Awadzi K, Basá̃ez MG, Biritwum N, Boakye D, Boatin B, et al. Onchocerciasis control: vision for the future from a Ghanaian perspective. Parasites Vectors. 2009;2:7. 10.1186/1756-3305-2-7 PMC2639371 19154624

[11] Rebollo MP, Zoure H, Ogoussan K, Sodahlon Y, Ottesen EA, Cantey PT (2018). Onchocerciasis: shifting the target from control to elimination requires a new first-step-elimination mapping. Int Health..

[12] Falling Rain Genomics Incorporated. Directory of cities and towns in the world [Internet]. Global Gazette version 2.1. 2019. p. Copyright 1996–2006. http://www.fallingrain.com/world/. Accessed 28 Oct 2020.

[13] Lamberton PHL, Cheke RA, Walker M, Winskill P, Osei-Atweneboana MY, Tirados I (2014). Onchocerciasis transmission in Ghana: biting and parous rates of host-seeking sibling species of the *Simulium damnosum* complex. Parasites Vectors..

[14] Lamberton PHL, Cheke RA, Winskill P, Tirados I, Walker M, Osei-Atweneboana MY (2015). Onchocerciasis transmission in Ghana: persistence under different control strategies and the role of the simuliid vectors. PLoS Negl Trop Dis..

[15] Lamberton PHL, Cheke RA, Walker M, Winskill P, Crainey JL, Boakye DA (2016). Onchocerciasis transmission in Ghana: the human blood index of sibling species of the* Simulium damnosum* complex. Parasites Vectors..

[16] World Health Organization. Onchocerciasis Guidelines for Stopping Mass Drug Administration and verifying Elimination of Human onchocerciasis Criteria and Procedures. Geneva: World Health Organization; 2016. http://www.who.int/onchocerciasis/resources/9789241510011/en/. Accessed 28 Oct 2020. 26913317

[17] Yaméogo L (2008). Special intervention zones. Ann Trop Med Parasitol..

[18] Biritwum NK, de Souza DK, Marfo B, Odoom S, Alomatu B, Asiedu O (2017). Fifteen years of programme implementation for the elimination of lymphatic filariasis in Ghana: impact of MDA on immunoparasitological indicators. PLoS Negl Trop Dis..

[19] Biritwum N-K, de Souza DK, Marfo B, Odoom S, Alomatu B, Asiedu O, et al. Fifteen years of programme implementation for the elimination of lymphatic filariasis in Ghana: impact of MDA on immunoparasitological indicators. PLoS Negl Trop Dis. 2017;11. 10.1371/journal.pntd.0005280 PMC5363798 28333930

[20] Noma M, Nwoke BEB, Nutall I, Tambala PA, Enyong P, Namsenmo A (2002). Rapid epidemiological mapping of onchocerciasis (REMO): its application by the African Programme for Onchocerciasis Control (APOC). Ann Trop Med Parasitol..

[21] Schulz Key H (1978). A simple technique to assess the total number of *Onchocerca volvulus* microfilariae in skin snips. Tropenmed Parasitol..

[22] Gyasi ME, Nsiire A, Yayemain D, Debrah OA, Asamani D, Gyapong J (2010). Trachoma in Northern Ghana: a need for further studies. Ophthalmic Epidemiol..

[23] Debrah O, Mensah EO, Senyonjo L, de Souza DK, Hervie TE, Agyemang D (2017). Elimination of trachoma as a public health problem in Ghana: providing evidence through a pre-validation survey. PLoS Negl Trop Dis..

[24] Le Berre R, Garms R, Davies JB, Walsh JF, Philippon B, Johnson CG (1979). Displacements of *Simulium damnosum* and strategy of control against onchocerciasis. Philos Trans R Soc Lond B Biol Sci..

[25] Baker RH, Guillet P, Sékétéli A, Poudiougo P, Boakye D, Wilson MD (1990). Progress in controlling the reinvasion of windborne vectors into the western area of the Onchocerciasis Control Programme in West Africa. Philos Trans R Soc Lond Ser B Biol Sci..

[26] Osei-Atweneboana MY, Eng JK, Boakye DA, Gyapong JO, Prichard RK (2007). Prevalence and intensity of *Onchocerca volvulus* infection and efficacy of ivermectin in endemic communities in Ghana: a two-phase epidemiological study. Lancet..

[27] Osei-Atweneboana MY, Awadzi K, Attah SK, Boakye DA, Gyapong JO, Prichard RK (2011). Phenotypic evidence of emerging ivermectin resistance in* Onchocerca volvulus*. PLoS Negl Trop Dis..

[28] Frempong KK, Walker M, Cheke RA, Tetevi EJ, Gyan ET, Owusu EO (2016). Does increasing treatment frequency address suboptimal responses to ivermectin for the control and elimination of river blindness?. Clin Infect Dis..

[29] African Programme for Onchocerciasis Control. Conceptual and operational framework of onchocerciasis elimination with ivermectin treatment. WHO/APOC. 2010. http://www.who.int/apoc/oncho_elimination_report_english.pdf Accessed 28 Oct 2020.

[30] World Health Organization. Criteria for certification of interruption of transmission/elimination of human onchocerciasis. Report of a meeting held in Geneva, 28–29 September 2000. WHO/CDS/CPE/CEE/2001.18a. Geneva; 2001. http://www.oepa.net/Documentos/CriteriosCertificacionOMS/WHO_CDS_CPE_CEE_2001.18a.pdf. Accessed 28 Oct 2020.

[31] World Health Organization. Report of the Third Meeting of the WHO Onchocerciasis Technical Advisory Subgroup, Geneva, 26–28 February 2019. Geneva; 2020. https://www.who.int/publications/i/item/9789240006638. Accessed 28 Oct 2020.

[32] Awadzi K, Edwards G, Opoku NO, Ardrey AE, Favager S, Addy ET (2004). The safety, tolerability and pharmacokinetics of levamisole alone, levamisole plus ivermectin, and levamisole plus albendazole, and their efficacy against* Onchocerca volvulus*. Ann Trop Med Parasitol..

