# Supplementary figures and images for: Onchocerciasis control in Ghana (1974–2016)
Source: Parasit Vectors. 2021 Jan 2;14:3. doi: 10.1186/s13071-020-04507-2 (PMC7778817; doi:10.1186/s13071-020-04507-2)

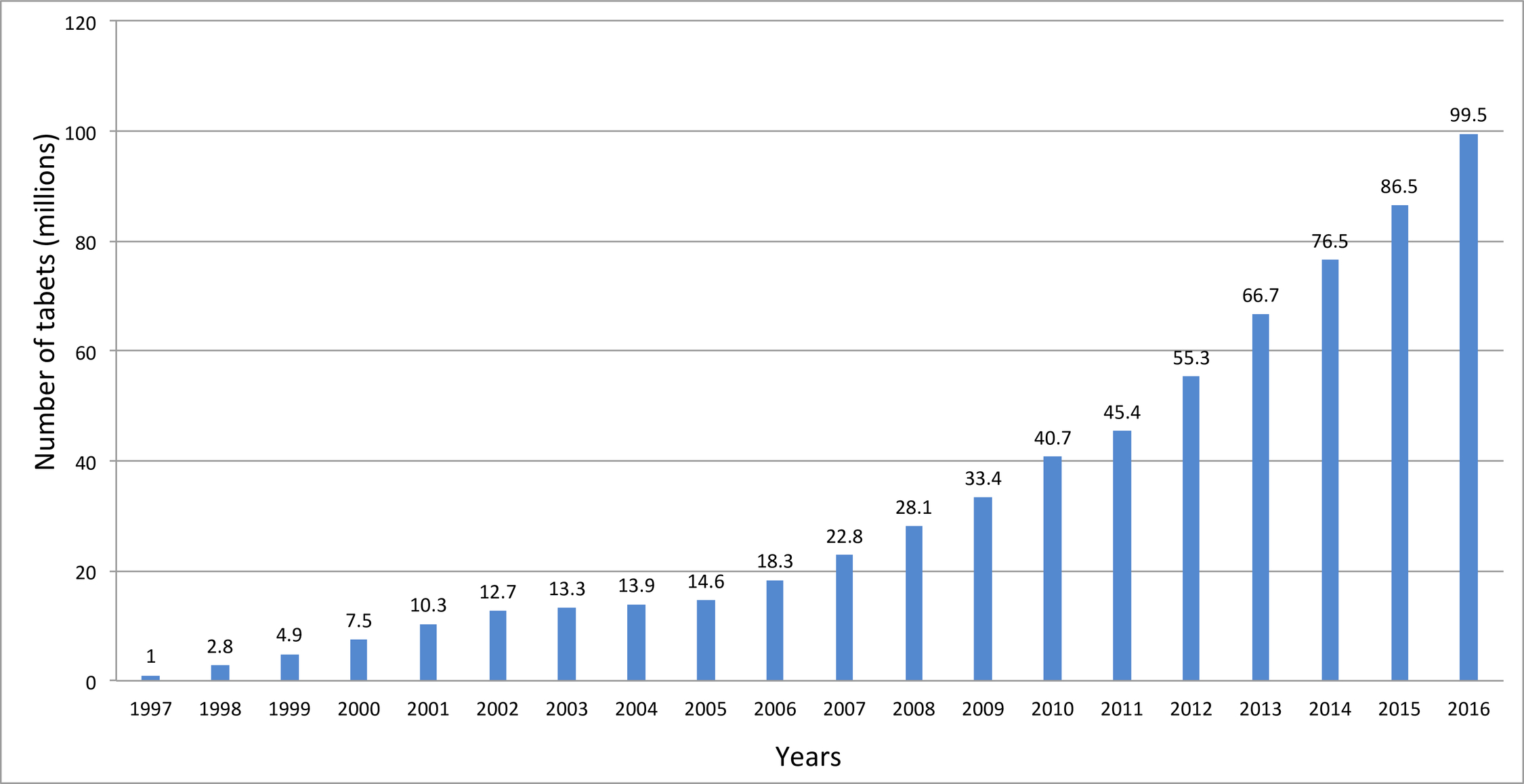

Supplement: Supplementary file 1 — Additional file 1: Fig. S1. Cumulative number of ivermectin tablets distributed (1997–2016). [file 13071_2020_4507_MOESM1_ESM.tif]

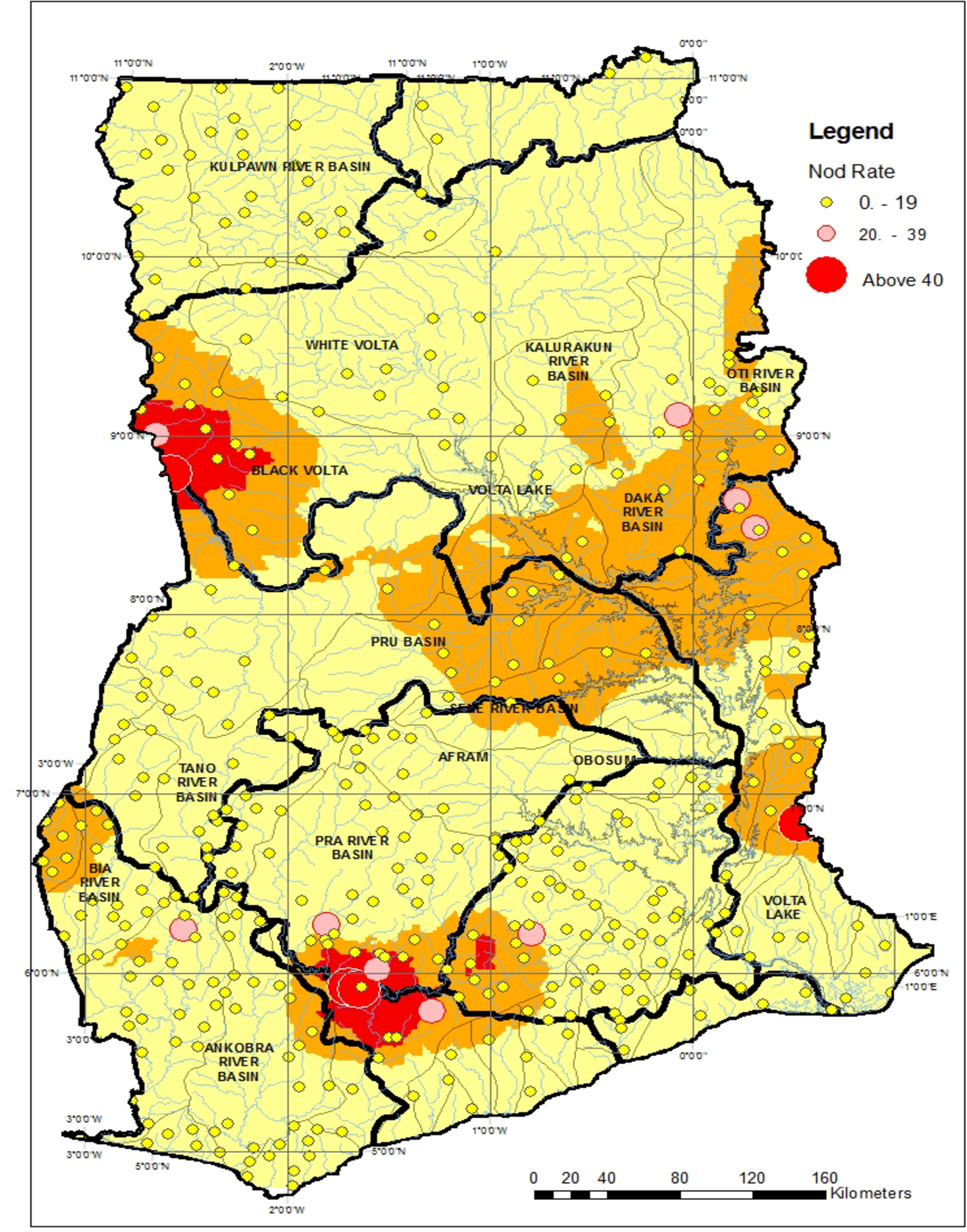

Supplement: Supplementary file 2 — Additional file 2: Fig. S2. Workforce at the community level for health intervention (2009–2016). [file 13071_2020_4507_MOESM2_ESM.tif]

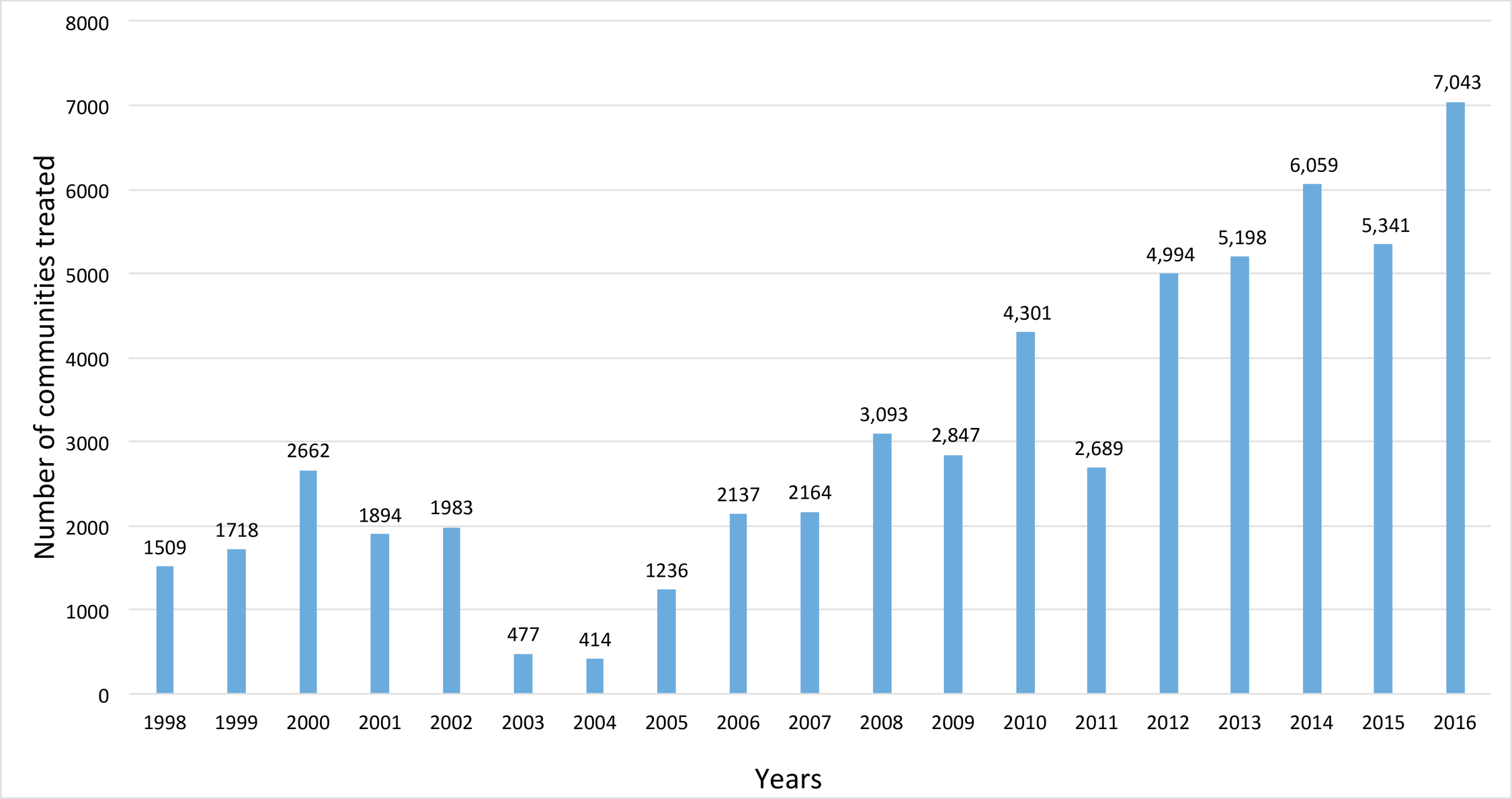

Supplement: Supplementary file 3 — Additional file 3: Fig. S3. Training of health workers (2009–2016). [file 13071_2020_4507_MOESM3_ESM.tif]

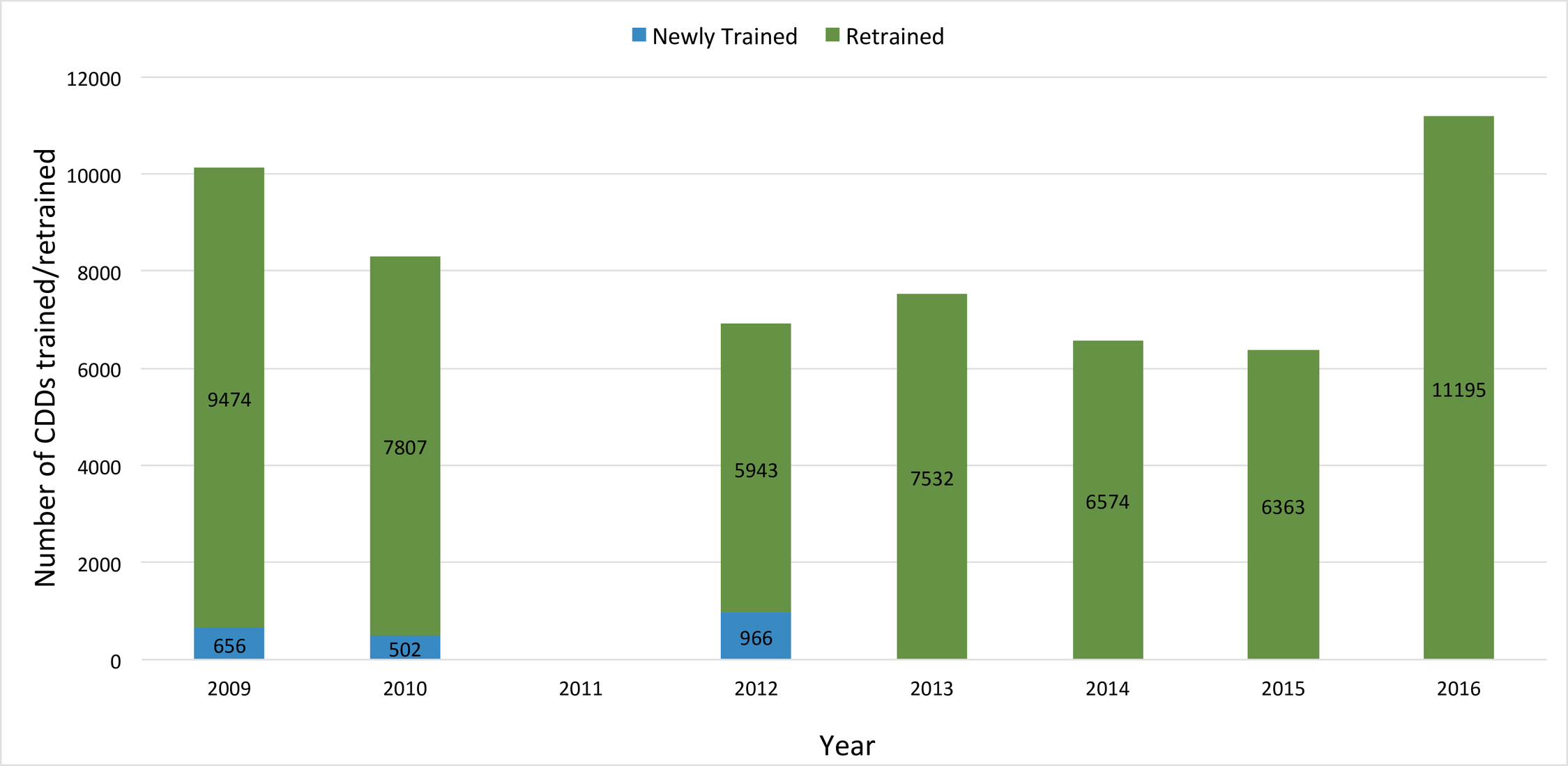

Supplement: Supplementary file 4 — Additional file 4: Fig. S4. Number of communities treated (1998–2016). [file 13071_2020_4507_MOESM4_ESM.tif]

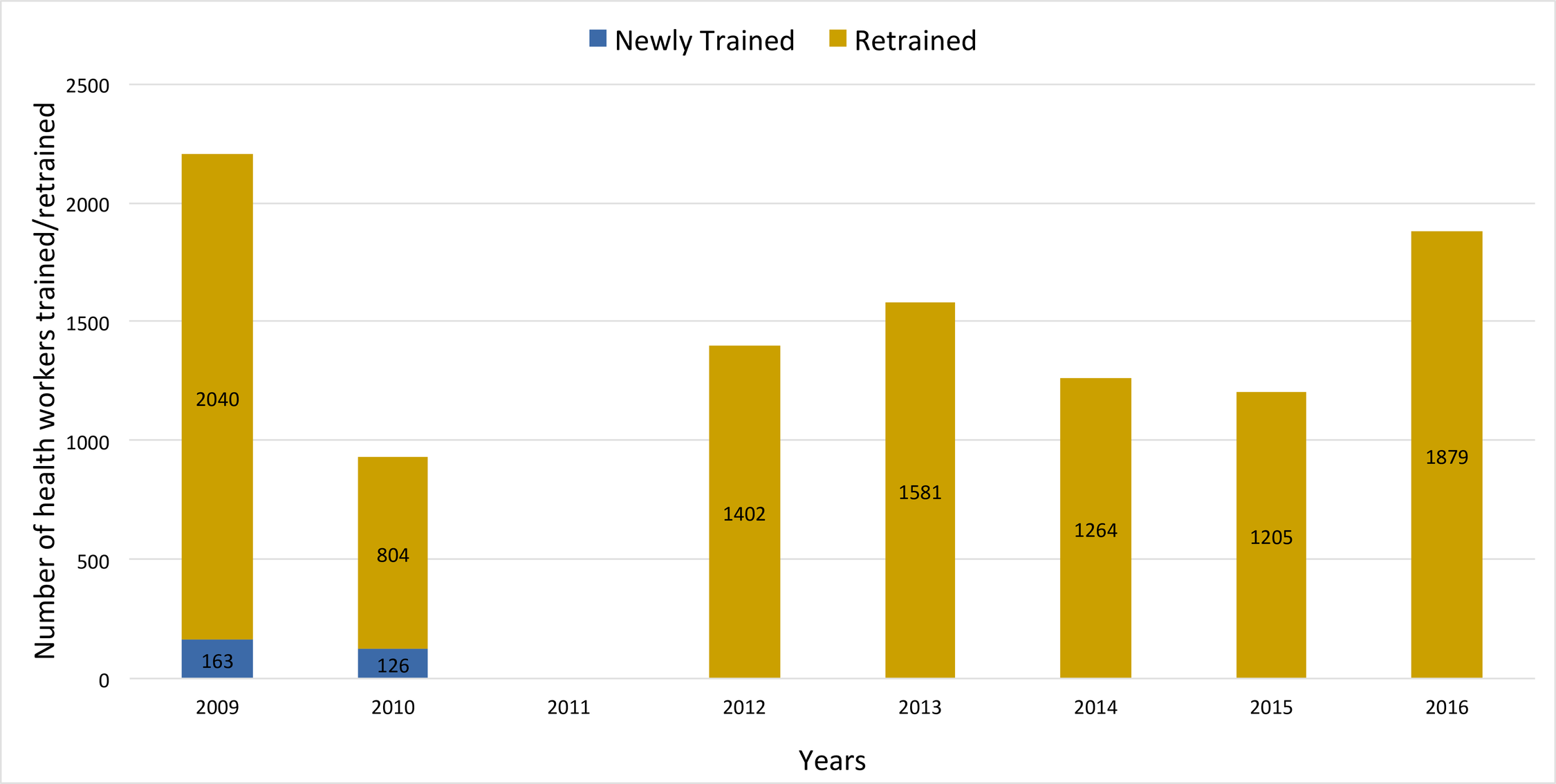

Supplement: Supplementary file 5 — Additional file 5: Fig. S5. REMO (2008) map identifying endemic areas for treatment. [file 13071_2020_4507_MOESM5_ESM.tif]
